# CIS4/403: Design and Implementation of an Intranet-based system for Real-Time Tele-Consultation in Oncology

**DOI:** 10.2196/jmir.1.suppl1.e9

**Published:** 1999-09-19

**Authors:** C Eccher, F Berloffa, F Demichelis, B Larcher, M Galvagni, A Sboner, A Graiff, S Forti

**Affiliations:** ^1^ITC-irst, Povo-TrentoItaly

**Keywords:** Intranet, Teleconsultation, Oncology

## Abstract

**Introduction:**

This study describes a tele-consultation system (TCS) developed to provide a computing environment over a Wide Area Network (WAN) in North Italy (Province of Trento), that can be used by two or more physicians to share medical data and to work co-operatively on medical records. A pilot study has been carried out in oncology to assess the effectiveness of the system. The aim of this project is to facilitate the management of oncology patients by improving communication among the specialists of central and district hospitals.

**Methods and Results:**

The TCS is an Intranet-based solution. The Intranet is based on a PC WAN with Windows NT Server, Microsoft SQL Server, and Internet Information Server. TCS is composed of native and custom applications developed in the Microsoft Windows (9x and NT) environment. The basic component of the system is the multimedia digital medical record, structured as a collection of HTML and ASP pages. A distributed relational database will allow users to store and retrieve medical records, accessed by a dedicated Web browser via the Web Server. The medical data to be stored and the presentation architecture of the clinical record had been determined in close collaboration with the clinicians involved in the project. TCS will allow a multi-point tele-consultation (TC) among two or more participants on remote computers, providing synchronized surfing through the clinical report. A set of collaborative and personal tools, whiteboard with drawing tools, point-to-point digital audio-conference, chat, local notepad, e-mail service, are integrated in the system to provide an user friendly environment. TCS has been developed as a client-server architecture. The client part of the system is based on the Microsoft Web Browser control and provides the user interface and the tools described above. The server part, running all the time on a dedicated computer, accepts connection requests and manages the connections among the participants in a TC, allowing multiple TC to run simultaneously. TCS has been developed in Visual C++ environment using MFC library and COM technology; ActiveX controls have been written in Visual Basic to perform dedicated tasks from the inside of the HTML clinical report. Before deploying the system in the hospital departments involved in the project, TCS has been tested in our laboratory by clinicians involved in the project to evaluate the usability of the system.

**Discussion:**

TCS has the potential to support a "multi-disciplinary distributed virtual oncological meeting". The specialists of different departments and of different hospitals can attend "virtual meetings" and interactively discuss on medical data. An expected benefit of the "virtual meeting" should be the possibility to provide expert remote advice from oncologists to peripheral cancer units in formulating treatment plans, conducting follow-up sessions and supporting clinical research.

